# Implantation of VVI pacemaker in a patient with dextrocardia, persistent left superior vena cava, and sick sinus syndrome: A case report

**DOI:** 10.1097/MD.0000000000006028

**Published:** 2017-02-03

**Authors:** Gongliang Guo, Lili Yang, Jinyi Wu, Liqun Sun

**Affiliations:** a Department of Cardiology, China-Japan Union Hospital, Jilin University; b Department of Gynecology and Obstetrics; c Department of Pediatric, the First Hospital of Jilin University, Changchun, Jilin Province, china.

**Keywords:** dextrocardia, persistent left superior vena cava, sick sinus syndrome, venous malformation, VVI pacemaker

## Abstract

**Background:**

Dextrocardia, or right-lying heart, is an uncommon congenital heart disease in which the apex of the heart is located on the right side of chest. Persistent left superior vena cava (PLSVA) is a rare venous anomaly that is often associated with the abnormalities of cardiac transduction system. A case with combination of dextrocardia, persistent left superior vena cava, and sick sinus syndrome has not been reported.

**Methods:**

We used different techniques including cardiac color Doppler echocardiography, 24-hour Holter monitoring, and abdominal ultrasound to make a diagnosis and treated the patient by implanting a VVI pacemaker.

**Results:**

A 50-year-old woman was admitted with a syncope. Angiography of the right atrium and superior vena cava, echocardiography, electrocardiography, and abdominal ultrasound revealed the presence of the combination of mirror image dextrocardia, PLSVA, and sick sinus syndrome. The complex structural anomalies presented great technical challenges for interventional treatments. After thorough examination and understanding of the structural anatomy and anomalies of the superior and inferior vena cava and cardiac chambers, we successfully treated this patient by implanting a VVI pacemaker.

**Conclusion:**

Physicians must be aware of the complexity of the morphological and anatomical structures of dextrocardia accompanying PLSVC. Given that the diagnosis of situs inversus was performed at a relatively advanced age, it is therefore important to make such a correct diagnosis followed by appropriate therapeutic intervention.

## Abstract 2

Supplemental Digital Content is available in the text

## 1 Introduction

Dextrocardia, that is right-lying heart, is a rare congenital heart defect in which the heart is predominantly localized on the right side of the thorax.^[1]^ The reported incidence of dextrocardia is 1 in 10,000 live births,^[2,3]^ and it accounts for 0.5% of cases of adult congenital heart disease.^[4]^ Mirror image dextrocardia, which is the most common subtype of dextrocardia, is often associated with other structural malformations.^[5]^ Persistent left superior vena cava (PLSVC) is also rare but is the most common systemic venous anomaly, with a reported incidence of 0.5% in the general population and accounting for 3% to 10% of cases of congenital heart defects.^[6]^ Cases of dextrocardia in combination with a PLSVC are extremely rare but have been previously reported and/or successfully treated. For example, Yener et al^[7]^ described a case of dextrocardia with PLSVC and a ventricular septal defect. Wan et al^[8]^ successfully treated a patient with dextrocardia, PLSVC, and ventricular tachycardia with a wearable defibrillator. Pott et al^[9]^ also successfully implanted a biventricular pacemaker in a patient with the combination of dextrocardia, PLSVC, and idiopathic dilated cardiomyopathy. Here, we present a rare case of dextrocardia and PLSVC (the right superior vena cava was normal) accompanied by sick sinus syndrome. This patient had a complicated venous anomaly, which posed a tremendous challenge for the completion of cardiac invasive procedures. We successfully treated this patient by implanting a VVI pacemaker. No similar case has been reported previously. We obtained approval from the Ethics Committee of Jilin University and a signed consent form from the patient for reporting this case.

## 2 Case presentation

**Figure 1:**
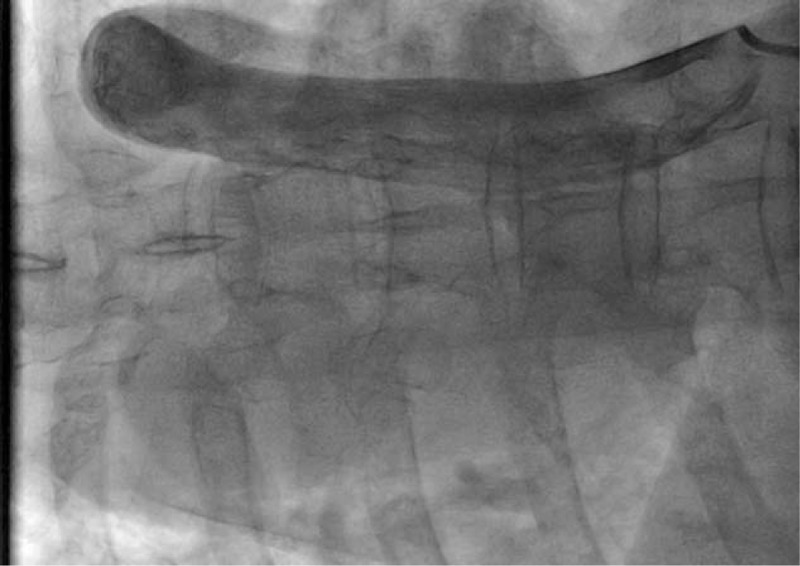
Angiography of the inferior vena cava revealed the malformation of inferior vena cava: inverted U-shape of inferior vena cava draining into the right atrium from the upper portion of the heart.

**Figure 2:**
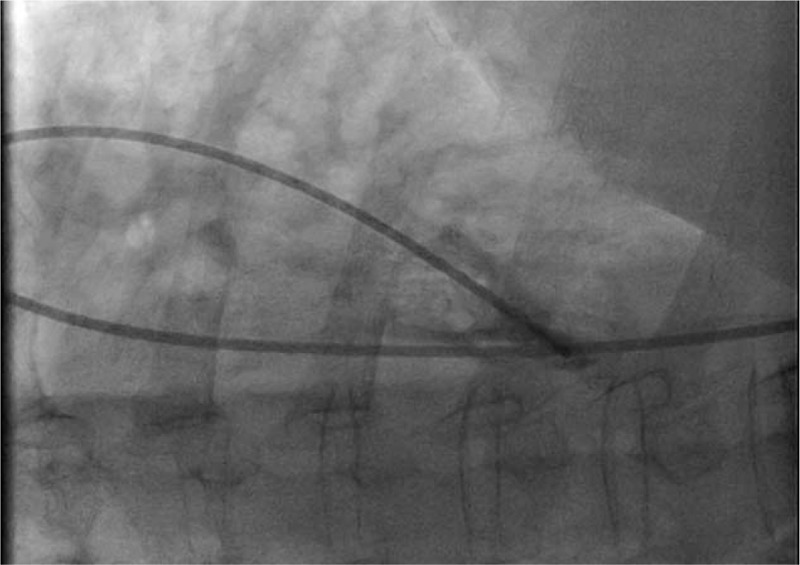
Right atrial angiography revealed the entrance of the catheter from the malformed inferior vena cava into the right atrium.

**Figure 3:**
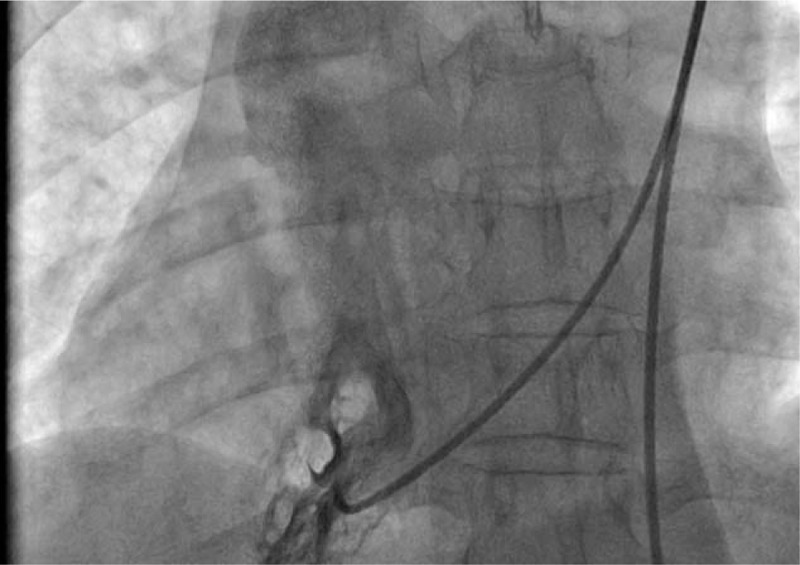
Angiographic image of the right ventricle. An inferior vena cava (IVC) catheter ran through the malformed inferior vena cava, right atrium, and tricuspid valve and entered the right ventricle.

**Figure 4:**
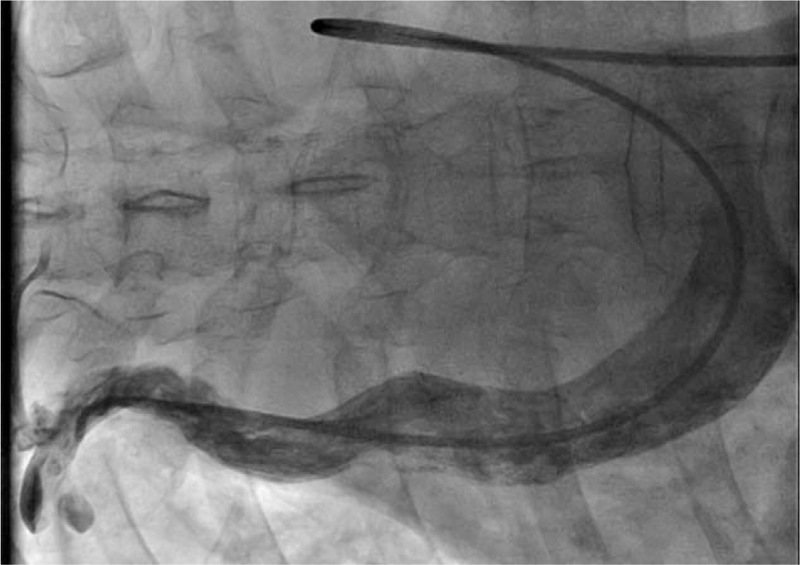
Angiographic image of the right superior vena cava. An inferior vena cava (IVC) catheter ran through the malformed inferior vena cava and right atrium and entered the right superior vena cava.

A 50-year-old female patient was admitted to our hospital because of a syncope 30 minutes ago and complaint of palpitations and malaise lasting for 1 year. Approximately 1 year before admission, the patient had started having palpitations and malaise, and a number of ECG examinations revealed sinus bradycardia with a heart rate of ∼45 beats per min (bpm) (Supplemental Fig. 1). At admission, physical examination showed a blood pressure of 150/80 mmHg, equal-sized pupils, and a heart rate of 43 bpm, and that apex beating was found in the right fifth intercostal space approximately 0.5 cm from the midclavicular line with no murmurs heard. Head computed tomography results were normal. ECG examination revealed that the morphology of waves in leads avR and avL were opposite to the normal, inverted P, QRS, and T waves in lead I, and that the graphics of the right-sided chest leads were similar to those of the left-sided ones. Cardiac color Doppler echocardiography examination revealed mirror-image dextrocardia. Twenty-four-hour Holter monitoring showed an average heart rate of 47 bpm, the longest PP interval of 3.31 seconds, nodal escape beats, and paroxysmal atrial fibrillation. Abdominal ultrasound examination revealed the liver and gallbladder were located on the left side and the spleen on the right. Based on the above examinations and clinical manifestations, we diagnosed this patient as having mirror image dextrocardia and sick sinus syndrome and designed the following surgical strategy to implant a ventricular pacemaker. Under local anesthesia, an insertion site was chosen on the right subclavian vein (because of dextrocardia, it was symmetrical with the conventional insertion site), and the guide wire was passed through the subclavian vein and superior vena cava. We expected that the guide wire would eventually reach the right ventricle. However, during the procedure, we found that the superior vena cava was very tortuous in its course and that the guide wire did not travel smoothly within the vessels and failed to reach the right ventricle. We therefore considered the existence of severe vascular malformations and aborted this attempt. To determine the possibility of the presence of vascular malformation, we performed right heart catheterization and venography through the femur and found a severe malformation of the inferior vena cava, in which the inferior vena cava did not directly drain into the right atrium from the bottom; instead, it formed an inverted U-shape and drained into the right atrium from the top (Fig. 1). Consistent with the diagnosis of dextrocardia, we found that the right atrium was located on the lower left section of the heart (Fig. 2), and that the right ventricle was located on the lower right section of the heart (Fig. 3). In addition, venography revealed that the right-sided superior vena cava also ran an abnormal, tortuous path (Fig. 4).

**Figure 5:**
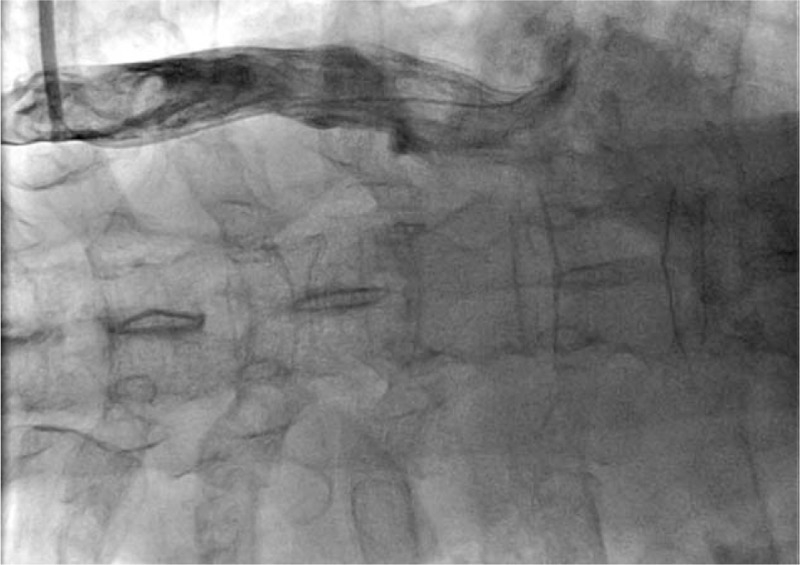
Image of the left superior vena cava obtained from atrial angiography performed via the left subclavian vein.

**Figure 6:**
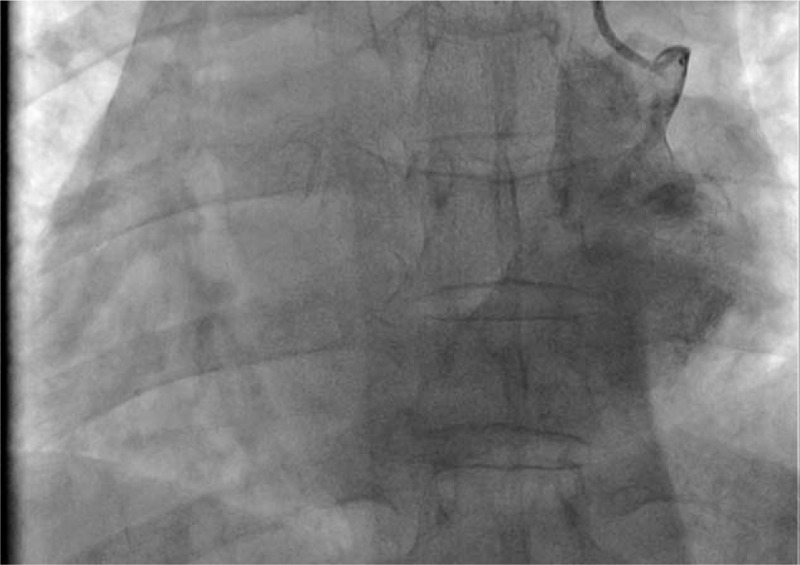
Angiographic image showing the drainage of the left superior vena cava into the right atrium.

After reviewing the angiographic images, we acknowledged that this patient had severe venous malformations and did not expect it would be possible to place the ventricle electrode via the right subclavian vein. To further understand the structural malformations of these veins and design a better approach for treatment, we once again performed a left subclavian venography and found the presence of PLSVA, in which the left superior vena cava directly drained into the right atrium via the coronary sinus (Figs. 5 and 6).

**Figure 7:**
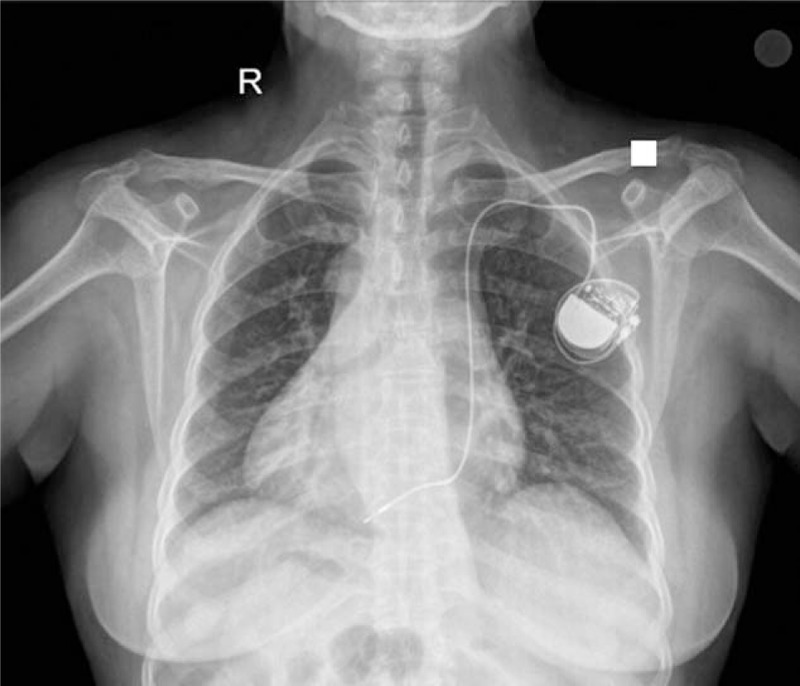
Chest X-ray demonstrating the correct placement of the VVI pacemaker.

After analysis of the angiographic results, we concluded that this patient had dextrocardia, PLSVA, and sick sinus syndrome. After obtaining a thorough understanding of the venous anatomy and anomalies of this patient, we successfully implanted a pacemaker lead in a proper location in the right ventricle through the left subclavian vein. The implanted VVI pacemaker had a pacing threshold of 0.6 V, impedance of 680 ohm, and ventricular sensing of 6.5 mV. Chest X-ray showed correct placement of the ventricular lead in the right ventricle (Fig. 7), and ECG after the treatment was normal (Supplemental Fig. 2).

## 3 Discussion

Dextrocardia is clinically characterized into 3 subgroups based on the position of heart chambers and abdominal organs.^[5]^ First is dextroversion (situs solitus or false dextrocardia): the heart is on the right side of the chest with the apex pointing to the right of the body. Dextroversion is often accompanied by other intracardiac anomalies and mainly caused by the positional shift and rotation of the right heart. Second one is dextroposition (situs ambiguous or isomerism): only the heart is on the opposite location but the other visceral organs are appropriately positioned. Dextroposition usually occurs when the anatomical abnormalities of lung, pleura, and/or diaphragm “push” the heart to the right side of the chest. Third is dextrocardia (situs inversus or mirror image dextrocardia): the anterior-posterior relationships of the different parts of the heart are normal, but the right-to-left orientation is reversed. Mirror image dextrocardia is the most common form of right-lying heart and is associated with different degrees of visceral transposition (i.e., situs inversus).^[5]^ The patient reported in this case was diagnosed with mirror-image dextrocardia.

PLSVC is also an uncommon congenital anomaly and may occur in isolation or in combination with other cardiac or vascular malformations.^[10]^ Based on the venous drainage sites, PLSVC may be classified into 4 types: type I, PLSVC drains into the right atrium through the coronary sinus; type II, PLSVC drains into the right atrium through the coronary sinus but has a connection with the left atrium, thus generating the right to left shunt; type III, PLSVC directly drains into the left atrium, resulting in a right to left shunt; and type IV, PLSVC is directly connected to the left pulmonary vein.^[11]^ Among these types of PLSVC, type I accounts for nearly 90% of all PLSVC cases.^[11]^ Although type I PLSVC usually does not have any significant hemodynamic consequences, a large amount of blood flows back to the atria through the coronary sinus vein and may subsequently dilate the sinus ostium, particularly in the absence of the right superior vena cava. Because the origins of the sinus node, atrioventricular node, and His-bundle are structurally close to the junction of the main veins and the coronary sinus, the incidence of arrhythmias in patients with PLSVC is also higher. The patient reported here had a type I PLSVC in combination with sick sinus syndrome.

Clinically, mirror-image dextrocardia in combination with PLSVC is extremely rare. A search of the Pubmed database using key words “dextrocadia,” and “persistent left superior vena cava” generated only a few publications,^[7–9,12–15]^ and another search of the same database using key words “dextrocadia,” “persistent left superior vena cava,” and “sick sinus syndrome” produced zero citations. To the best of our knowledge, we are the first to report a case of successful implantation of a VVI pacemaker via a malformed left superior vena cava in a patient with mirror-image dextrocardia combined with PLSVC and sick sinus syndrome. The complex anatomical structures and rare combination of dextrocardia and PLSVC posed great challenges for the surgical intervention. In this particular case, the major challenge was determining how to smoothly guide the electrode into the right ventricle via tricuspid valve. To achieve this goal, we performed angiography of the right atrium and right ventricle and precisely determined the positions of the right atrium, right ventricle, and tricuspid valve. We also performed venography to accurately identify the venous malformations. After obtaining a thorough knowledge of the structural malformations, we appropriately adjusted the plasticity of the guidewire and safely passed the electrode through the tricuspid valve, which was finally anchored in the correct position within the right ventricle. Given the severe inferior vena cava malformation and complex positions of atria and ventricles of this patient, it would be difficult for a temporary pacemaker to reach its designated site. This patient had an uneventful recovery after surgery.

In conclusion, we report here a rare case of dextrocardia combined with PLSVC and sick sinus syndrome, which was successfully treated with the implantation of a VI pacemaker after thorough examination and understanding of the structural anatomy and abnormalities of the veins and heart chambers. We are publishing this case to share our clinical experience for use in the diagnosis and treatment of similar cases in the field. It has to be pointed out that there is no criterion standard to follow in treating patients who bear dextrocardia with PLSVC. Physicians must be aware of the complexity of the morphological and anatomical structures of dextrocardia accompanying PLSVC, and a lack of knowledge may render additional difficulty with therapeutic interventions and pose serious risks. In addition, we can maximally diminish the risk of complications and improve the success rate of cardiac invasive approaches only after we fully control therapeutic techniques and their respective appropriate applications.

## Supplementary Material

Supplemental Digital Content
