# Supplementary material for: Implantation of VVI pacemaker in a patient with dextrocardia, persistent left superior vena cava, and sick sinus syndrome: A case report
Source: Medicine (Baltimore). 2017 Feb 3;96(5):e6028. doi: 10.1097/MD.0000000000006028 (PMC5293471; doi:10.1097/MD.0000000000006028)

Supplemental Figure 1: Electrocardiograph of the patient at admission

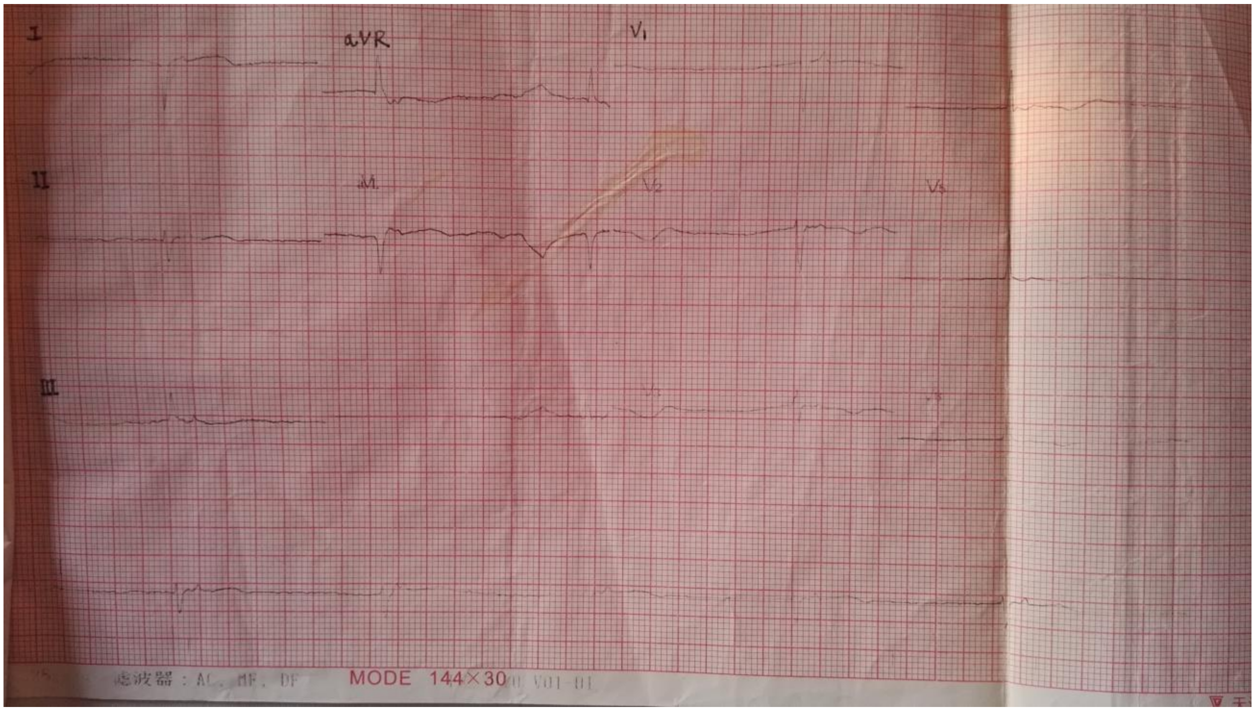

Supplemental Figure 2: Current electrocardiograph of the patient after treatment.

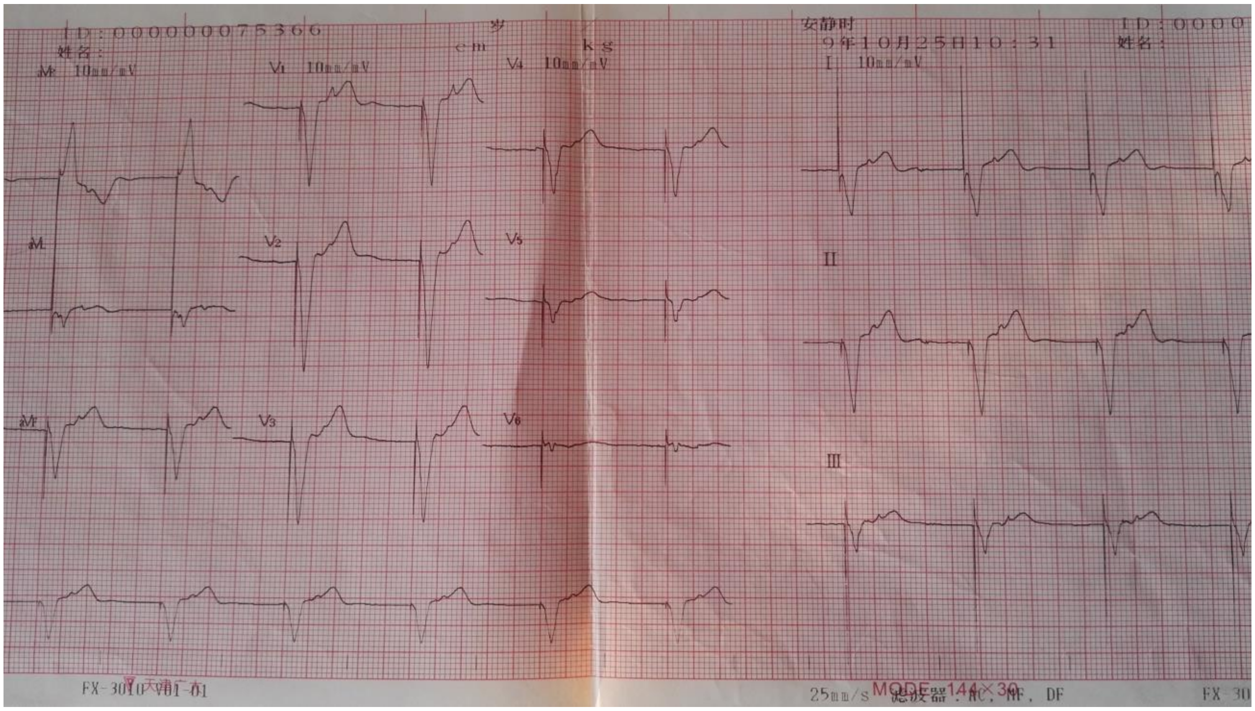

Supplement: Supplemental Digital Content [file medi-96-e6028-s001.pdf]
